# Homœopathic Medical School in Buffalo

**Published:** 1878-04

**Authors:** 

**Affiliations:** Uptonville, N. Y.


UPTONVILLE, N. Y., March 9, 1878.

*Editors Buffalo Medical and Surgical Journal.*

GENTLEMEN: Some one has kindly called my attention to the proceedings of the State Legislature, in which I see that a bill has been introduced incorporating a Homœopathic College in your city. Allow me to congratulate you upon this acquisition to the *scientific* (?) institutions of your commonwealth. One of the incorporators of the institution is, I learn, the author of a talented (?) exposition of Modern Homœopathy, which was read before the Medical Association of Buffalo some time since. The writer of the article in question kindly sent me one of the "reprints" of his paper, which I read with considerable interest, but owing to the absence of my book of familiar quotations I could not verify the origin of all his statements. It struck me, however, that a deal of good paper could have been saved, beside considerable labor of compiling on the part of the writer of the answer to the question, "What is Modern Homœopathy?" if he had ended his paper with the apt and concise remark which he attributes to his old preceptor: "its a d—d humbug." By the way, Messrs. Editors, what is supposed to constitute a reprint? I have not had the pleasure of seeing the number of the *National Observer* containing the article on "What is Modern Homœopathy," but after reading the pamphlet sent me I naturally had some curiosity to see what the Editors of that journal might say of the article, and seeing a copy of the *Journal* for January on the office table of a medical friend, I hastened to scan its pages. Imagine my surprise when I learned from it that the *reprint* which I had received in *December* would not be *printed* until the *February* issue of the journal. One or two other questions: Does the Buffalo Medical Association have any control over the papers read before it? Can an essayist without permission allow a paper read before it to be printed in a Homœopathic journal? Was such permission given in this case? As I understand the rule, all papers read before medical or scientific societies are the property of such societies, and it is a glaring breach of courtesy at least for the authors to print the papers in any other way than in the proceedings of the society, with the announcement that they were read before the society, without first gaining consent of the organization.

But I have wandered far from the original intention of this letter, and perhaps might have done well to follow the example set by your *Journal* and the Buffalo Medical Association, in utterly ignoring the "reprint."

I am very much gratified that my predictions on reading the paper are verified, and that the astute author has taken up his bed and walked into the camp of his friends, the "Modern Homœopaths." After his vain and verbose attempt to befoul his own nest, the regular profession are well rid of him. A man who cannot find liberty enough in the ranks of the regular medical profession of to-day to prescribe any medicine on the earth in the air, or in the waters under the earth, in any dose which pleases—be it large or small—does not and should not belong in the ranks of that profession. The liberty which he is panting for is bounded by the narrow horizon of his own vision, aud is more correctly spelled and pronounced "notoriety."

When the Homœopathic College is founded please inform me of the corps of instructors which are in the future to make Buffalo the centre of light upon that elusive, deceptive, and enigmatical science (?) "Modern Homœopathy."

Yours, "NIL DESPERANDUM."

